# Passing the test

**DOI:** 10.1242/bio.20136940

**Published:** 2013-10-30

**Authors:** Rachel Hackett, O. Claire Moulton, Jordan W. Raff

It was while drafting an editorial on the timely subject of publishing ethics that it first came to light that *Biology Open* (*BiO*) was one of the journals that received as a submission a spoof article devised by a journalist working for the journal *Science* (Bohannon, 2013). The article was described as being obviously flawed to any competent scientist. 304 spoof articles were submitted to Open Access (OA) journals worldwide, including *BiO*: at the time the *Science* report was published, 157 of the journals had accepted the paper and 98 had rejected it. Reassuringly, the handling editor at *BiO* had editorially rejected the article.

There has been much discussion of this ‘sting’ within the science and publishing community. In particular, several commentators have highlighted and questioned why only OA journals were tested in this way; there was no control to test the rate of acceptance of the spoof paper in non-OA journals, even though these findings have been (and were perhaps intended to be) interpreted by some as proof that OA publishing is in some way inherently flawed.

Although *BiO* seems to have passed some sort of test (and, in fact, has a rejection rate of about 50%), no journal can afford to be complacent. The soundness of *BiO*'s peer review process and associated ethical practices will continue to be a key focus of its editors, and now seems a good time to reiterate some of the principles that guide our editorial policy.

## Peer review at *BiO*

Unbiased, independent, critical assessment is of vital importance in scholarly publishing (Raff, 2013). *BiO* aims to provide rapid peer-reviewed publication of scientifically sound observations and conclusions. Reviewers are asked to confirm that the experimental work is properly conducted and that the conclusions are adequately supported by the data. We do not require any assessment of the significance, relative importance or impact of a paper, as we believe this will gradually emerge post-publication. Therefore, we ask reviewers to simply address the following criteria:

- The experimental research component is technically and ethically sound.
- The conclusions are all adequately supported by the data.
- The title and the summary of the manuscript accurately report the main conclusions.
- The figures and tables are clear.
- The language is clear and accessible.

We are currently implementing the addition of a new acceptance criterion – that the methods used are described in sufficient detail to allow proper repetition of the experiments. Recent articles have highlighted failures in the reliability and reproducibility of published research (e.g. http://www.nature.com/nature/focus/reproducibility) and we believe that it is important to tackle this issue.

## Associated ethical practices

Over the past decade or so, many initiatives and codes of practice have been established within the scientific publishing community. Many now feel routine (such as having dotted lines between spliced gel lanes), whereas others continue to evolve. The Company of Biologists is a member of COPE, The Committee on Publication Ethics (http://publicationethics.org). When the decision was made to launch *BiO*, it was the COPE guidelines and codes of practice that were employed. In addition, *BiO* follows the International Committee of Medical Journal Editor's (http://www.icmje.org) recommendations for the “conduct, reporting, editing and publication of scholarly work in medical journals”.

Among the many important practices that have been instituted is the publication of Conflict of Interest and Author Contribution statements. The policies briefly described below have been devised in conjunction with our academic partners to ensure the accurate, timely, fair and ethical peer review and publication of scientific papers submitted to *BiO*.

### 

#### Image manipulation and fraud

Adjustment of digital images with computer software is acceptable. However, the final image must remain representative of the original data. *BiO* checks figures for evidence of image manipulation (Rossner and Yamada, 2004). In addition, the corresponding author is required to certify at submission that they are aware of all manipulations performed on any digital images that accompany their article.

#### Competing interests disclosure

A competing or conflict of interest is anything that might (or might be perceived to) inappropriately influence (bias) the full and objective presentation, review or publication of research findings. Competing interests can be financial, professional or personal, and can be held by authors, their employers, sponsors of the work, reviewers, Editors and editorial staff.

*BiO* requires that a Conflict of Interest statement be included in each article, even if this is used to declare that there are no conflicts. Authors must include information regarding the provider of financial and material support of their research in the Funding section. Reviewers, Editors and Editorial staff are also required to disclose financial or professional associations that might interfere with their objectivity.

#### Author contributions

Recent discussions have focused on the evolving issue of authorship. *BiO* requires that authors include a statement in the manuscript that specifies the contributions of each author. An author is someone who has made significant and substantial contributions to a study. These should include conception, design, execution and interpretation of the data being published, and preparing the article.

The ICMJE has recently proposed a new criterion for authorship. This states that an author must “be accountable for all aspects of the work in ensuring that questions related to the accuracy or integrity of any part of the work are appropriately investigated and resolved. In addition to being accountable for the parts of the work he or she has done, an author should be able to identify which co-authors are responsible for specific other parts of the work. In addition, authors should have confidence in the integrity of the contributions of their co-authors.” This has prompted discussion of the genuine feasibility of this, in particular with regards to large multi-centre research.

The *BiO* submission process is set by default to ensure that all authors are included in relevant editorial correspondence. Papers must be submitted with the agreement of all authors, and all authors must approve the version to be published. However, this can't always protect the journal from those intent on perpetrating misconduct, such as using fake but active and plausible email addresses for co-authors who may not be aware that an article has been submitted in their name – to use one actual example.

Despite the policies, checks and balances that have been implemented, it can be difficult to identify and block papers that include flawed work or outright fraudulent data. Although BiO passed the spoof paper ‘test’, we will continue to review our role in maintaining the integrity of the scientific record.
